# The voice characterisation checklist: psychometric properties of a brief clinical assessment of voices as social agents

**DOI:** 10.3389/fpsyt.2023.1192655

**Published:** 2023-07-21

**Authors:** Clementine J. Edwards, Oliver Owrid, Lucy Miller, Hassan Jafari, Richard Emsley, Mar Rus-Calafell, Thomas K. J. Craig, Moya Clancy, Hamish McLeod, Miriam Fornells-Ambrojo, Jeffrey McDonnell, Alice Montague, Mark Huckvale, Sandra Bucci, Gillian Haddock, Philippa Garety, Thomas Ward

**Affiliations:** ^1^Institute of Psychiatry, Psychology and Neuroscience, King’s College London, London, United Kingdom; ^2^South London and Maudsley NHS Foundation Trust, London, United Kingdom; ^3^Mental Health Research and Treatment Centre, Faculty of Psychology, Ruhr-Universität Bochum, Bochum, Germany; ^4^University of Glasgow, Glasgow, United Kingdom; ^5^NHS Greater Glasgow & Clyde, Glasgow, United Kingdom; ^6^University College London, London, United Kingdom; ^7^North East London NHS Foundation Trust, London, United Kingdom; ^8^School of Health Sciences, Faculty of Biology, Medicine and Health, Manchester Academic Health Sciences Centre, University of Manchester, Manchester, United Kingdom; ^9^Greater Manchester Mental Health NHS Foundation Trust, Manchester, United Kingdom

**Keywords:** psychosis, voice-hearing, characterisation, phenomenology, auditory hallucination

## Abstract

**Aim:**

There is growing interest in tailoring psychological interventions for distressing voices and a need for reliable tools to assess phenomenological features which might influence treatment response. This study examines the reliability and internal consistency of the Voice Characterisation Checklist (VoCC), a novel 10-item tool which assesses degree of voice characterisation, identified as relevant to a new wave of relational approaches.

**Methods:**

The sample comprised participants experiencing distressing voices, recruited at baseline on the AVATAR2 trial between January 2021 and July 2022 (*n* = 170). Inter-rater reliability (IRR) and internal consistency analyses (Cronbach’s alpha) were conducted.

**Results:**

The majority of participants reported some degree of voice personification (94%) with high endorsement of voices as distinct auditory experiences (87%) with basic attributes of gender and age (82%). While most identified a voice intention (75%) and personality (76%), attribution of mental states (35%) to the voice (‘What are they thinking?’) and a known historical relationship (36%) were less common. The internal consistency of the VoCC was acceptable (10 items, α = 0.71). IRR analysis indicated acceptable to excellent reliability at the item-level for 9/10 items and moderate agreement between raters’ global (binary) classification of more vs. less highly characterised voices, κ = 0.549 (95% CI, 0.240–0.859), *p* < 0.05.

**Conclusion:**

The VoCC is a reliable and internally consistent tool for assessing voice characterisation and will be used to test whether voice characterisation moderates treatment outcome to AVATAR therapy. There is potential wider utility within clinical trials of other relational therapies as well as routine clinical practice.

## 1. Background

Voice-hearing, or auditory verbal hallucinations (AVH), are a common experience among those diagnosed with psychotic disorders (1) and there is growing interest in voice-hearing across diagnoses as well (2). While voices can occur in the general population without associated distress (3, 4), for a significant number of voice-hearers, the experiences become persecutory, debilitating and persist despite interventions (5).

Voices are often described in terms of an experience of communication with a personified other (6, 7), and there has been longstanding interest in this aspect of voice phenomenology (8, 9). Personification or characterisation of voices (terms we view as essentially equivalent) is common, and around 70% of voice-hearers associate their voice(s) with ‘characterful qualities’ (10); that is, people or person-like entities with distinct characteristics, such as gender, age, patterned emotional responses, or intentions. In a study involving people accessing early intervention in psychosis services 40% of participants described complex voice personification (6). This was defined as the voice having more than one kind of person-like quality, including elaborate descriptions of intentional states (the voice wants/thinks/feels), agency (the voice will ‘make something happen’), or identity (the voice ‘comes’ from somewhere or has a specific and idiosyncratic ontological status). The increased recognition of the communicative and relational aspects of voice-hearing demonstrated by such studies, reflects an important evolution from early information processing accounts which centred on the misattribution of an ‘auditory stimulus’ to an external source [see (11) for a discussion]. While existing tools adopt a multidimensional approach to voices, including assessment of coping strategies, rating of beliefs, and acceptance or mindfulness, there are currently no validated measures assessing voice characterisation (12).

There is growing interest in developing treatments, which are tailored to diverse phenomenological features of voice-hearing (13). This includes a new wave of psychological interventions which target the relationship between the person and their voice, specifically Relating Therapy (14), Talking with Voices (15), and AVATAR therapy (16). In AVATAR therapy, a novel therapeutic context allows ‘face-to-face’ dialogue between the person and a computerised representation of their persecutory voice. Using voice-transformation software, the therapist facilitates a dialogue between the person and the avatar in which the person develops an increased sense of power, control, and confidence within the relationship. This approach has been shown, in a fully powered trial, to reduce voice frequency and voice-related distress when compared with an active control at the end of therapy (primary endpoint) although group differences did not persist at follow-up (17). A large multi-site randomised controlled trial focused on optimization and implementation is underway (18). While there is promising evidence of effectiveness, including emerging replication by independent research teams (19) there is a need for research into factors which might influence AVATAR therapy outcomes that are likely to be relevant to other relational approaches.

A study published as part of the first AVATAR therapy trial investigated whether the experience of a person’s dominant voice as a highly characterised social agent was associated with differences in voice engagement in both daily life and during AVATAR therapy (20). In line with study hypotheses, more highly characterised voices were associated with increased behavioural engagement with voices in daily life and, crucially, increased dialogic engagement during AVATAR dialogues. While this suggested that voice characterisation may be an important factor in engagement with AVATAR therapy, the study was not designed to test the key question as to whether this phenomenological aspect of voices might moderate treatment outcomes. To date, studies exploring voice characterisation or personification have utilised coding of phenomenology based on detailed clinical assessments (20) or qualitative interviews (6). This approach is well suited to exploration of what can be complex and nuanced voice phenomenology but presents challenges in a large clinical trial with the requirement for a comprehensive assessment battery of validated measures.

A tool capable of assessing voice characterisation in an efficient but robust manner is therefore required to examine the impact of voice characterisation on outcomes following intervention. Such a tool would also have wider utility beyond the research context, for example, as an aid to comprehensive clinical assessment of this hitherto neglected aspect of the voice hearing experience. The AVATAR2 trial is a multi-site randomised controlled trial of AVATAR therapy in comparison to treatment as usual (18). As part of the trial design, we have developed the Voice Characterisation Checklist (VoCC) based on the framework developed in AVATAR1 (20) and aim to examine its reliability with the large sample of voice-hearers taking part in AVATAR2. This group of voice-hearers report current voice-related distress and include a wide range of pathways to care and voice-hearing experiences.

### 1.1. Aims

- To examine the reliability and factor structure of the Voice Characterisation Checklist (VoCC) in a sample of people who hear distressing voices.
- To report a preliminary description of the characterisation of the voice-hearing experiences in participants in the AVATAR2 clinical trial.

## 2. Methods

### 2.1. Recruitment

AVATAR2 is a multi-site parallel group randomised controlled trial which is due to be completed in October 2023 (18). Randomisation to AVATAR-brief (six sessions), AVATAR-extended (12 sessions) therapy or Treatment as Usual was performed on a 1:1:1 allocation basis and was stratified by voice characterisation (more vs. less highly characterised). Four United Kingdom research sites took part in the trial: King’s College London, University College London, The University of Manchester and the University of Glasgow. Each research site was linked to two National Health Service (NHS) Trusts/Health Boards, where potential participants were identified and referred to the trial by their treating clinician. Self-referrals were considered too, and recruitment databases and consent for contact (C4C) initiatives were also utilised where available to maximise the participant pool.

The full inclusion and exclusion criteria can be found in the published protocol (18), in brief, participants were adults who had been hearing a distressing voice(/s) within the context of psychosis for at least 6 months at the time of the baseline assessment.

### 2.2. Procedure

The Voice Characterisation Checklist (VoCC) was administered as a semi-structured interview by research assistants as part of the baseline assessment which took place face-to-face or online. To prevent rater drift across the trial, research assistants received training, passed an observed assessment, and attended weekly group supervision from clinicians in administration of this and other measures.

### 2.3. Measures

#### 2.3.1. Voice characterisation checklist

**Figure 1:**
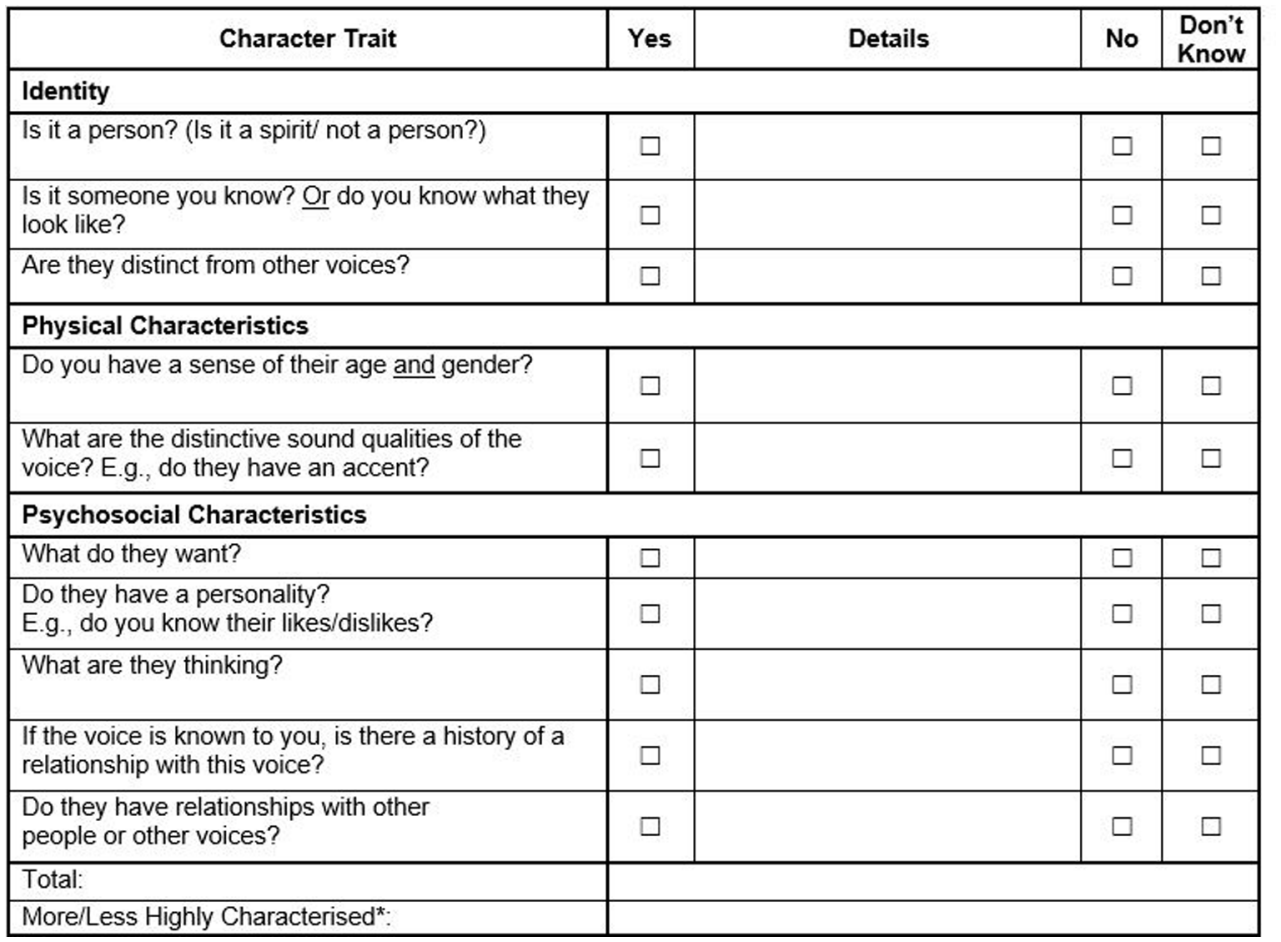
Voice characterisation checklist (VoCC).

The voice characterisation checklist was devised from a qualitative coding framework employed by Ward et al. (20) in their study of voice characterisation and avatar engagement, which was itself informed by previous phenomenological work, e.g. (10). The VoCC is administered as an interview and scored by the interviewer, the language used to refer to the voices is flexible to enhance communication and understanding and interviewers may use a variety of terms; singular, plural, voices and others. In the VoCC there are 10 items, scored ‘Yes’, ‘No’ or ‘Do not Know’ which assess key areas highlighted in the qualitative coding framework: identity, physical and psychosocial characteristics. Items are scored ‘Yes’ where participants can provide information in response to the question, a ‘No’ where they have no information to provide, and ‘Do not Know’ if they are unsure if it applies to their voice. Anecdotally reported time to administer the VoCC ranged from 5 to 30 min. The range of scores is 0–10 and a score of 7+ is the threshold for a more highly characterised voice as this ensures the voice has traits in all three categories. The VoCC is free to use and available in Figure 1.

### 2.4. Statistical tests

The descriptive statistics of the included sample as well as the frequency of VoCC responses were reported, to provide a general overview of the data. The scale’s reliability was assessed through inter-rater reliability and internal consistency analysis (Cronbach’s alpha). Inter-rater reliability was assessed in a sample of 33 AVATAR2 participants, who were randomly selected from the pool of participants’ IDs across four sites: South London (*n* = 8), North London (*n* = 8), Manchester (*n* = 9), and Glasgow (*n* = 8). A total of 13 research assistants from the four sites are represented in the scores used. The lead author (CE), acted as the expert scorer and blind rated the VoCC from audio recordings. Internal consistency, on the other hand, was determined by assessing the correlation between items within the scale.

To determine the underlying construct or factors and assess the validity of the conceptual model, an exploratory factor analysis (EFA) was conducted on the 10 VoCC items (21). For this analysis, the iterated principal axis method, also known as principal factors, was used as the factoring estimation method. This method is a robust and efficient way of finding the few factors that account for the common variance of several variables. Oblique rotation (promax) was used to better interpret the factor loading (22). Promax allows for correlated factors, which is more realistic in many psychological studies (23).

Before conducting the factor analysis, the Bartlett test of sphericity was conducted. A value of *p* less than 0.05 indicates that the correlation matrix of the observed variables is not an identity matrix, and that the variables are correlated enough, therefore suitable for factor analysis. Additionally, the Kaiser-Meyer-Olkin (KMO) measure of sampling adequacy was calculated to provide an overall measure of the overlap (shared variance) between the variables. A KMO value of more than 0.6 is generally considered acceptable, indicating that the sample is suitable for factor analysis (24) [Statistical analyses were conducted using Stata Statistical Software: Release 17. College Station, TX: StataCorp LLC, and R statistical programme (2022) (25)].

## 3. Results

### 3.1. Sample

The sample comprised participants who had completed their baseline assessment as part of the AVATAR2 trial between January 2021 and July 2022, the cut-off date for uploading the database for this study (*n* = 170). All participants, demographic characteristics are presented in Table 1.

**Table 1:** Demographic characteristics.

Demographic characteristic	Overall (*N* = 170)
Age	
Mean (SD)	37.9 (12.9)
Median [Min, Max]	36.0 [18.0, 70.0]
Gender	
Male	100 (58.8%)
Female	67 (39.4%)
Other	3 (1.8%)
Ethnicity	
White	103 (60.6%)
Black Caribbean	13 (7.6%)
Black African	12 (7.1%)
Black-Other	4 (2.4%)
Indian	5 (2.9%)
Pakistani	8 (4.7%)
Chinese	1 (0.6%)
Other	24 (14.1%)
Highest level of schooling	
Primary school	1 (0.6%)
Secondary no exams qualifications	10 (5.9%)
Secondary (O/CSE equivalent)	34 (20.0%)
Secondary (A level equivalent)	28 (16.5%)
Vocational Education/college	48 (28.2%)
University degree/professional qualification	49 (28.8%)
Roughly how old were you when you first started hearing voices?	
Mean (SD)	29.5 (75.7)
Median [Min, Max]	21.0 [3.00, 999]
Primary ICD-10 diagnosis	
F20—Schizophrenia	79 (46.5%)
F32.3—Severe depressive episode with psychotic symptoms	12 (7.1%)
F22—Persistent delusional disorders	1 (0.6%)
F23—Acute and transient psychotic disorders	2 (1.2%)
F24—Induced delusional disorder	1 (0.6%)
F25—Schizoaffective disorders	14 (8.2%)
F28—Other nonorganic psychotic disorders	4 (2.4%)
F29—Unspecified nonorganic psychosis	49 (28.8%)
F31—Bipolar affective disorder	4 (2.4%)
Missing	4 (2.4%)

### 3.2. Frequency of responses

**Figure 2:**
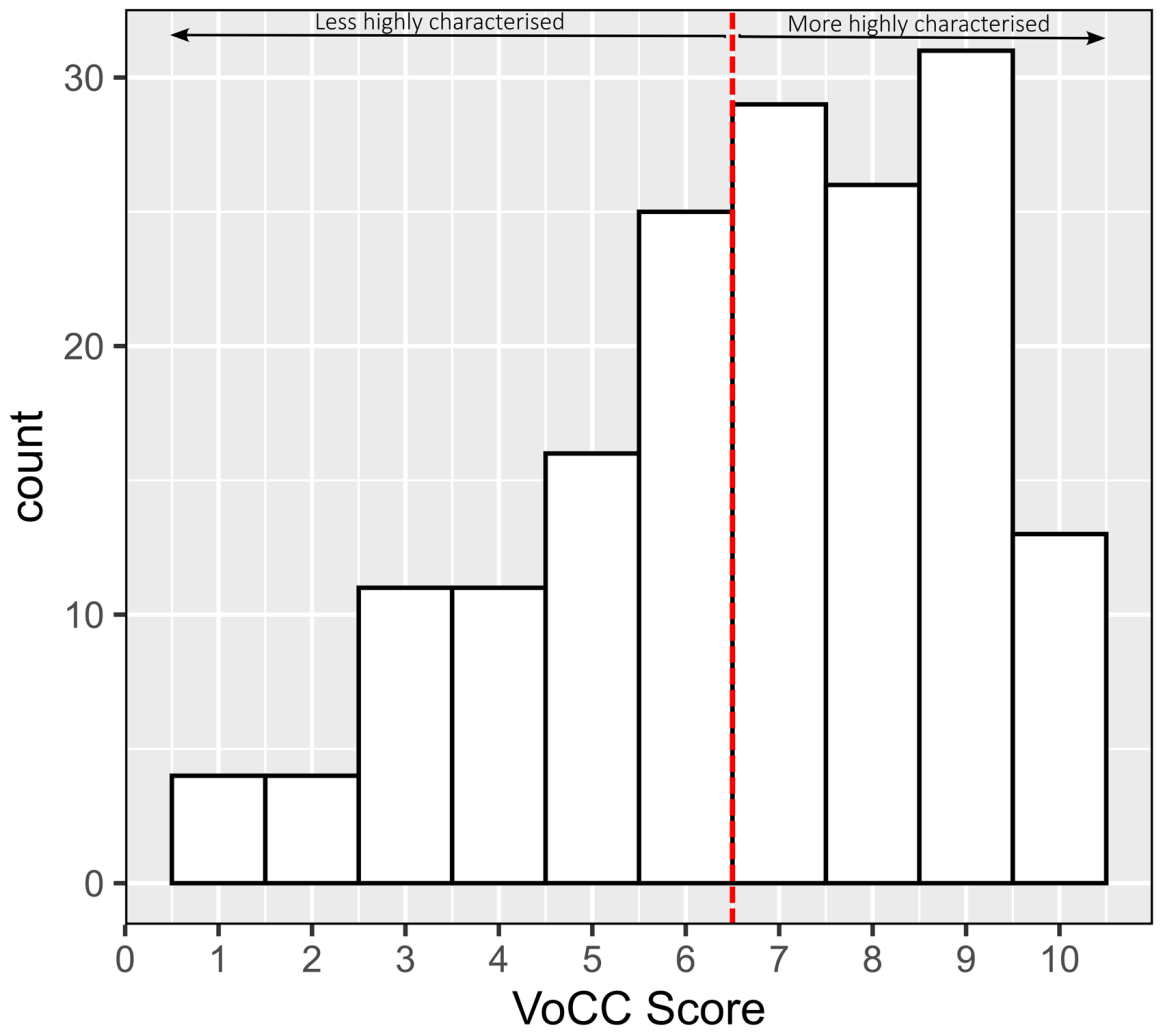
The histogram of VoCC overall score for 170 participants.

The ‘Unclear/Do not Know’ response choice is recoded as ‘Absent’ to create a dichotomised variable. The frequency of dichotomised response choices for each item is presented in Table 2 and Figure 2. Overall, there are 561 Absent (33%) and 1,139 Present responses (67%). Bases on the overall cut-off score 7 or higher, from the 170 participants, 71 (41.8%) were classified as less highly characterised and 99 (58.2%) were classified as more highly characterised, with the ratio of 1.4 (more/less).

**Table 2:** Voice characterisation checklist items.

Item		Description	Present		Absent	
			Freq.	%	Freq.	%
Identity	1	Is it a person? Or is it a spirit?	159	94%	11	6%
	2	Is it someone you know? Or do you know what they look like?	109	64%	61	36%
	3	Are they distinct from other voices?	148	87%	22	13%
Physical Characteristics						
	4	Do you have a sense of their age and gender?	140	82%	30	18%
	5	What are the distinctive sound qualities of the voice? e.g., do they have an accent?	121	71%	49	29%
Psychosocial characteristics						
	6	What do they want?	128	75%	42	25%
	7	Do they have a personality? e.g., do you know their likes/dislikes?	130	76%	40	24%
	8	What are they thinking?	59	35%	111	65%
	9	If the voice is known to you, is there a history of a relationship with this voice?	62	36%	108	64%
	10	Do they have relationships with other people or other voices?	83	49%	87	51%

An example of responses to the VoCC for more versus less highly characterised voices can be seen in Table 3. These responses were given by two participants of the AVATAR2 trial when administered the VoCC at baseline assessment, details have been altered to protect patient identity.

**Table 3:** Example responses to the VoCC.

	More highly characterised	Less highly characterised
Is it a person?	Yes they have got a name and everything. I have got a very distinct idea of who it is… so the leader is a guy called Bill he lives below me apparently. He lives there with his wife but now he is changed that to his partner because he is now bisexual. He threatens to beat me up constantly but he is a coward because whenever I say yes okay let us do this he will not meet up with me to do it so he is basically a loudmouth who just swears and rants and raves and he is the most unpleasant out of all of them.	I think it is… I have never asked this question to myself so I do not know. I think it might be like… it is not a person as such. I think it is more, maybe, I do not really believe in ghosts but it might be a spirit or a bad entity.
Age and gender	Yeah I had say he is about 40.	No.
Distinctive sound qualities	Well it was a Scottish guy initially and then the Scottish guy seemed to morph into Bill and now Bill sounds more Irish than Scottish.	There is no accent. It is almost like my thoughts but it is saying words and sentences.
What does the voice want?	He wants my money basically and also to punish me. The idea as well is that they will get me sectioned, somehow take my flat off me—I do not know how they will do that—then they will get a tenant and charge them rent.	I am not sure I have never asked it.

### 3.3. Statistical analysis

To evaluate the item-to-item relationship of the VoCC, a pairwise correlation analysis was conducted on the 10 binary variables (indicating the presence or absence of each characteristic). The results of this analysis are presented in Table 4. Subsequently, an exploratory factor analysis was performed on this matrix to identify underlying latent factors and patterns of association among the variables. The highest correlation observed was between the presence of Q2 and Q9 (*r* = 0.49), while the lowest correlation was found between the presence Q2 and Q6 (*r* = −0.002).

**Table 4:** Correlation matrix across VoCC items.

	Q1	Q2	Q3	Q4	Q5	Q6	Q7	Q8	Q9	Q10
Q1	1									
Q2	0.302***	1								
Q3	0.112	0.260***	1							
Q4	0.255***	0.297***	0.235**	1						
Q5	0.202**	0.282***	0.142	0.285***	1					
Q6	0.071	−0.002	−0.099	0.200**	0.117	1				
Q7	0.136	0.250**	0.117	0.325***	0.229**	0.229**	1			
Q8	0.091	0.133	0.134	0.240**	0.082	0.274***	0.259***	1		
Q9	0.150	0.490***	0.110	0.255***	0.212**	0.066	0.161*	0.115	1	
Q10	0.257***	0.314***	0.201**	0.329***	0.154*	0.178*	0.237**	0.227**	0.287***	1

^***^p < 0.001, ^**^p < 0.01, and ^*^p < 0.05.

#### 3.3.1. Factor analysis

The Bartlett sphericity test findings were acceptable (*Chi^2^* = 238.9, *df* = 45, *p* < 0.0001) and KMO = 0.772 (>0.60 is desirable). Two factors had an eigenvalue of more than one and cumulatively explained about 29% of the data variance. The correlation between the two factors was 0.63 and the factor loading for each item is presented in Table 5.

**Table 5:** Factor loading for the two explored factors after promax (oblique) rotation.

Item	Item description	Factor I	Factor II
Q2	Is it someone you know? Or do you know what they look like?	0.892	
Q9	If the voice is known to you, is there a history of a relationship with this voice?	0.575	
Q3	Are they distinct from other voices?	0.379	
Q10	Do they have relationships with other people or other voices?	0.366	
Q1	Is it a person? (Is it a spirit/not a person?)	0.364	
Q4	Do you have a sense of their age and gender?	0.348	
Q5	What are the distinctive sound qualities of the voice? e.g., do they have an accent?	0.343	
Q6	What do they want?		0.665
Q8	What are they thinking?		0.484
Q7	Do they have a personality? e.g., do you know their likes/dislikes?		0.413

#### 3.3.2. Internal consistency

The α coefficient (Cronbach’s α) for the 10 items of the VoCC was 0.71, which is considered acceptable within the range of 0.7–0.8. An examination of item-level correlations and Cronbach’s α after removing each item revealed no significant impact on the overall α coefficient, as none of the coefficients exceeded the all-items coefficient (Table 6).

**Table 6:** Item level internal consistency.

Item	Item-test correlation	Item-rest correlation	Alpha (item removed)
Q1	0.42	0.32	0.69
Q2	0.64	0.48	0.66
Q3	0.38	0.24	0.70
Q4	0.63	0.51	0.66
Q5	0.51	0.34	0.69
Q6	0.39	0.21	0.71
Q7	0.56	0.41	0.68
Q8	0.50	0.32	0.69
Q9	0.56	0.39	0.68
Q10	0.62	0.45	0.67
VoCC			0.71

#### 3.3.3. Inter-rater reliability

The agreement among reviewers was measured using three coefficients: percentage agreement, Cohen’s Kappa, and Krippendorff’s Alpha. The levels of agreement were categorised as follows: poor (0), slight (0.1–0.2), fair (0.21–0.4), moderate (0.41–0.6), substantial (0.61–0.8), or near perfect (0.81–0.99) (26). The inter-rater coefficients were measured first for each of the items (Table 7) and then for the overall categorisation (more vs. less highly characterised; Table 8). At the item-level, inter-rater reliability showed acceptable to excellent reliability for Q1, Q2, Q3, Q4, Q5, Q6, Q8, Q9, and Q10 with coefficients ranging from (Cohen’s Kappa = 0.61–1.0) and poor reliability for Q7 (Cohen’s Kappa = 0.40). The inter-rater reliability for overall categorisation was in the moderate range.

**Table 7:** Inter rater reliability.

Item	Agreement	Cohen’s Kappa	Krippendorff’s Alpha
Q1	100.00	1.00	1.00
Q2	84.85	0.67	0.67
Q3	93.94	0.72	0.72
Q4	93.94	0.76	0.77
Q5	84.85	0.61	0.61
Q6	93.94	0.82	0.82
Q7	78.79	0.40	0.41
Q8	81.82	0.63	0.62
Q9	87.88	0.73	0.73
Q10	96.97	0.94	0.94

Number of subjects = 33.

**Table 8:** The inter-rater agreement between the two raters’ VoCC categorisation (more vs. less).

	Coefficient	[95% Conf. Interval]
Percent agreement	0.788	0.640–0.935
Cohen’s Kappa	0.549	0.240–0.859
Krippendorff’s Alpha	0.556	0.245–0.866

Number of subjects = 33, Ratings per subject = 2.

## 4. Discussion

This study aimed to present the VoCC as a novel brief (10 items) tool for assessing the extent to which a distressing voice is experienced as a characterised social agent. The study has demonstrated its reliability and internal consistency within a large sample of people who experience distressing voices, recruited as part of the AVATAR2 trial. The findings therefore establish the VoCC as a useful research tool, capable of reliably (and quickly) assessing voice characterisation, which we hypothesise to be a potential moderator of treatment outcome in AVATAR therapy. In addition to use in a research context, where the VoCC’s brevity means it is easily integrated as part of an assessment battery, the tool has also been designed with wider utility in mind as a means of facilitating assessment of voice characterisation in routine clinical practice.

The descriptive data indicate that most people in the AVATAR2 sample report voices which are personified to some degree (94%) with high endorsement of voices as distinct auditory experiences (from one another and other sounds; 87%) and with associated basic attributes of gender and age (82%). Endorsement of psychosocial aspects was more varied. For example, while most people identified a basic voice intention (75%) and personality (76%), only around a third (35%) endorsed the item assessing attribution of mental states to the voice (‘What are they thinking?’). A similar minority of people identified a known historical relationship with the voice (36%) although the nature of these autobiographical relationships was not possible to determine from the checklist-context, which is likely to be crucial within the nuance of a relational intervention, where developmental trauma often plays a pivotal role. This descriptive pattern of endorsement across items was supported by the factor analysis which confirmed two factors, one incorporating physical and identity characteristics, and the other the psychosocial characteristics. The two items focused on relationships between the voice and others (Q9 and 10), originally conceptualised as psychosocial characteristics, loaded onto Factor I. The stronger association between these relational items and the identity and physical characteristics of the voice rather than the psychological items in Factor II should be examined in further validation of this scale. Overall, the findings are consistent with the proposition that characterisation (or personification) is a common feature of voice-hearing but also suggest the relevance of potential ‘levels of agency’ (27). While not designed to explore the granular complexity of voice agency, the data from the VoCC appear broadly consistent with earlier phenomenological work (6) suggesting that most voices recurred over time, had a distinct character, but could not be related to a known person (termed ‘internally individuated agency’) (27) and reported by 75% of people in the study by Alderson-Day et al. (6).

In summary, the findings presented here therefore confirm, in a large empirical/quantitative study, that voice characterisation is a common phenomenon among distressed voice hearers, with most of this sub-sample endorsing the items regarding physical characteristics and identity. Fewer people (although still a significant minority of 30–40%) endorsed the psychosocial items around the intention and thoughts of the voice, which may reflect more general difficulties in mental state attribution (28). The threshold for more highly characterised voices in the VoCC (a score of 7 or above) requires someone to endorse items across both the physical and psychosocial categories. This does not account for the complexity of the characteristics, but only that an awareness of both physical and psychosocial components are part of the person’s experience of the voice; this therefore is a low threshold for considering a voice to be more highly characterised when compared with the thresholds devised utilising qualitative frameworks. In line with this, we found 58.2% people reached the threshold for more highly characterised voices in this sub-sample compared to earlier work (20) in which 33% percent reported high voice characterisation, 42% medium and 25% low. Previous work (6, 20) highlight differences in voice engagement between high characterisation versus low/medium characterisation meaning that the current VoCC threshold will require further validation in future work. Nonetheless, from a clinical utility standpoint, the VoCC presented in this paper appears a useful tool to facilitate clinical assessment around this potentially important feature of voice-hearing (see clinical implications).

### 4.1. Limitations

While we have demonstrated reliability and internal consistency, validity of the VoCC was not examined because, to our knowledge, there are no validated quantitative measures which assess this specific construct. Future studies could explore convergent validity of the VoCC with coding of voice personification based on qualitative analysis, e.g. (6). It should be noted that the purpose of the VoCC is not to supplant the valuable insights delivered through qualitative work but rather to connect this important phenomenological work with the exigencies of a clinical trial and routine clinical practice. With respect to constructs which are plausibly linked to characterisation, the DAIMON measure (29) has been developed to assess the dialogical and emotional aspects of the relationship(s) between the voice-hearer and their voices and relationships with the VoCC could be explored in future research.

While reliability of the categorisation of voices as more versus less highly characterised was acceptable overall, the least reliable question from the item-level analysis was ‘does the voice have its own personality?’ While this might be viewed as a central question, assessing a sense of personality or character is arguably a more complex task compared to other items. It may therefore be that this item is less suited to a briefer ‘checklist’ with evidence that rater disagreement related to times where researchers were rating based on contextual information emerging at other stages of the assessment. It was notable that the overall reliability of the measure was improved with removal of this item. Therefore, one suggested option is to streamline the VoCC to include nine items but retain this question at the end as an optional (but suggested) aid to clinical assessment.

Finally, it is important to note that participants in this study (*n* = 170) were recruited as part of a trial for a relational intervention for voices (AVATAR therapy), so we are not able to generalise these findings to people who hear voices more generally, both in clinical groups and people who experience voices without an associated need for care.

### 4.2. Future directions

The VoCC was developed as part of the AVATAR2 trial, to enable voice characterisation to be included as a moderator of treatment outcome following AVATAR therapy. The VoCC has been used to stratify randomisations according to degree of voice characterisation (adopting a binary classification of ‘more highly’ vs. ‘less highly’ characterised). The tool has been suitable for integration within a comprehensive trial baseline assessment and the findings are positive with respect to establishing reliability and internal consistency. However, linked to its use as a stratification variable, a further key test of utility of the VoCC will come in the planned analysis of moderation of treatment outcome by degree of characterisation. If the VoCC does show utility with respect to these planned moderation analyses, it would suggest opportunities for exploring its use in trials of other relational approaches to working with distressing voices. For example, the Talking with Voices approach adopts an inclusion criterion based on people experiencing voices which are (at least to some degree) dialogic in form, given the nature of the therapy which involves direct (facilitated) dialogues with the voices. This inclusion decision is based on a discussion with participants to establish whether the approach is a ‘good fit’ for the person. Pilot work in the Talking with Voices approach suggests that instances in which people were unable or unwilling to engage in voice dialogue were relatively uncommon (15). Nonetheless, if characterisation as assessed by VoCC is shown to moderate treatment outcome to AVATAR therapy, it would be of interest to explore whether this is also observed in other dialogical approaches.

In addition to use in clinical trials, the questions themselves have been reported as helpful by some participants on the AVATAR2 trial, underscoring the importance of routinely assessing the social and relational elements relevant to the person and their voices. In our view, this relates to an attitude of respectful curiosity to voice phenomenology and developmental context which is central to the AVATAR therapy approach. We recommend potential use of the VoCC in clinical practice as part of a standard voices assessment. Use of the tool delivers an important, early message that the clinician is respectfully open to considering voices as nuanced, social communicative agents within the person’s life rather than just a symptom. A richer understanding of voice characterisation, including attribution of thought and intention, can facilitate the process of building understanding and meaning making. It also acts as an invitation to consider possible mirroring of current voice experiences with other relationships, autobiographical context, and the role of trauma (See also (15)). Future work using the VoCC could also benefit from measurement of potentially related constructs such as theory of mind, paranoia and expressivity.

## 5. Summary

This study has, for the first time, presented a brief tool to assess degree of voice characterisation (the VoCC), which is reliable, internally consistent, and capable of being delivered as part of clinical research and practice. The VoCC meets a need for robust measures to assess constructs relevant to relational therapies. Moving forward, the key test of utility will be whether it is helpful in helping us understand the question of whether certain forms of voice-hearing are more amenable to dialogical interventions such as AVATAR therapy.

## Data availability statement

The original contributions presented in the study are included in the article/supplementary material, further inquiries can be directed to the corresponding author.

## Ethics statement

The studies involving human participants were reviewed and approved by NHS Health Research Authority London—Camberwell St Giles Research Ethics Committee. The patients/participants provided their written informed consent to participate in this study. Written informed consent was obtained from the individual(s) for the publication of any potentially identifiable images or data included in this article.

## Author contributions

CE, TW, PG, MR-C, and TC designed the VoCC measure. CE, TW, LM, OO, HJ, RE, and PG designed the evaluation study of the VoCC. CE, OO, LM, HJ, RE, MR-C, TC, MC, HM, MF-A, JM, AM, MH, SB, GH, PG, and TW are members of the clinical trial management committee, which oversees conduct of the trial and data collection, and reviewed the manuscript and contributed to the interpretation of the analysis. HJ and RE conducted the analysis. CE, TW, HJ, OO, and LM drafted the manuscript. All authors contributed to the article and approved the submitted version.

## Funding

This study is funded by The Wellcome Trust Ltd., through an Innovations Project award (grant reference [215471/Z/19/Z]). The funding body has no role in the design of the study or the collection, analysis, and interpretation of data or the writing of the manuscript. The work was also part funded by the National Institute for Health and Care Research (NIHR) Maudsley Biomedical Research Centre at South London and Maudsley NHS Foundation Trust and King’s College London (PG and RE). RE is supported by an NIHR Research Professorship (NIHR300051). MR-C acknowledges individual funding from the Sofja Kovalevskaja Award (Alexander von Humbold Foundation and Ministry of Education and Research, Germany). GH acknowledges individual funding from an NIHR Senior Investigator Award (NIHR201393). SB is supported by an NIHR Research Professorship (NIHR300794) and is Director and shareholder of CareLoop Health Ltd., a spin out from the University of Manchester to develop and market digital solutions for remote monitoring using smartphones for mental health conditions, currently schizophrenia and postnatal depression. SB also reports research funding from The Wellcome Trust.

## Conflict of interest

The authors declare that the research was conducted in the absence of any commercial or financial relationships that could be construed as a potential conflict of interest.

## Publisher’s note

All claims expressed in this article are solely those of the authors and do not necessarily represent those of their affiliated organizations, or those of the publisher, the editors and the reviewers. Any product that may be evaluated in this article, or claim that may be made by its manufacturer, is not guaranteed or endorsed by the publisher.

## Author disclaimer

The views expressed are those of the author(s) and not necessarily those of the NIHR or the Department of Health and Social Care.
